# An Assay to Evaluate the Function of Liposomal Platelet Substitutes Delivered to Platelet Aggregates

**DOI:** 10.3389/fbioe.2019.00077

**Published:** 2019-04-12

**Authors:** Suyun Janet Tan, Keiko Nakahara, Keitaro Sou, Shinji Takeoka

**Affiliations:** ^1^Department of Life Science and Medical Bioscience, Graduate School of Advanced Science and Engineering, TWIns, Waseda University, Tokyo, Japan; ^2^Research Institute for Science and Engineering, Waseda University, Tokyo, Japan

**Keywords:** platelet aggregation, platelet substitutes, fluorescence, imaging techniques, microscopy, liposomes, nanoparticles

## Abstract

Aggregation of liposomal platelet substitutes with activated platelets is the primary endpoint to estimate hemostatic potential. Although light transmission aggregometry is a “gold standard” in assessing platelet aggregation *in vitro*, this method is less specific and sensitive when tested using liposomal platelet substitutes. In the current study, a new method is developed to evaluate the function of platelet substitutes. By labeling liposomes with a fluorescent dye, DiD, we evaluated their ability to target platelet aggregates using a fluorescence microscope. By incorporating an image-based 96 microtiter microplate, this method was optimized by varying the final lipid concentrations and washing times and validated using unmodified liposomes (e.g., L550 with 0 mol% of carboxylic headgroup lipid; L551 with 9 mol% of carboxylic headgroup lipid) and modified liposomes (e.g., H12-L551 with 9 mol% of carboxylic headgroup lipid and 0.3 mol% of dodecapeptide). Our results showed that 200 μM of H12-L551 liposomes and four washes represent optimal conditions for quantitative fluorescence imaging. This method allowed users to qualitatively observe the fluorescently labeled liposomes involved in platelet aggregates. The imaging analysis tool was sufficiently sensitive to quantitatively determine the significantly enhanced delivery of the modified liposomes to platelet aggregates. This enhancement was achieved using dodecapeptide, which specifically binds to activated platelets. This robust and high-throughput method enables the evaluation of liposome function and should facilitate the development of platelet substitutes with a greater ability to target platelet aggregates.

## Introduction

Platelets are blood components which play a central role in the physiological and pathological processes of hemostasis and thrombosis (Jurk and Kehrel, 2005). Platelets are the smallest biological cells in whole blood (~0.5 × 3.0 μm) (Hartwig et al., 1999), and through their involvement in hemostasis are crucial in preserving life after an injury that results in significant bleeding. Platelets form a hemostatic plug on injured blood vessels to prevent excessive bleeding through primary and secondary hemostasis. This process includes platelet adhesion, platelet secretion, platelet aggregation, microvesicle formation, and clot retraction/stabilization (McRedmond et al., 2004; Jurk and Kehrel, 2005). With the aim of identifying the responsible proteins and finding the underlying mechanisms of diseases of platelet function, many hematologists and researchers have developed an interest in studying the protein-protein interactions that occur in platelet sensing and responding pathways. Two major key proteins that have been identified are fibrinogen (Fbg) and glycoprotein IIb/IIIa (GPIIb/IIIa) for platelet aggregation.

GPIIb/IIIa has been shown to act as a Ca^2+^-dependent heterodimer, which serves as a receptor for Fbg or specifically binds to the histidine residues 400 and 401 of Fbg at the surface of activated platelets (Fitzgerald and Phillips, 1985; Rivas and Gonzalez-Rodriguez, 1991). Activation of the Fbg receptor, a function of GPIIb/IIIa after platelet stimulation, involves a conformational change through “inside out” signaling, while “outside in” signaling facilitates the recruitment of resting platelets to the growing thrombus by platelet activation (Li et al., 2010). Therefore, Fbg or very specifically, dodecapeptide (HHLGGAKQAGDV, H12) derived from a fibrinogen γ chain carboxyl-terminal sequence recognizes the active form of GPIIb/IIIa on the activated platelet membrane. During platelet stimulation, platelet agonists such adenosine diphosphate (ADP) are secreted from platelet granules that act via G protein-coupled receptors to reinforce GPIIb/IIIa-dependent platelet aggregation (Jurk and Kehrel, 2005).

We have demonstrated that a liposomal drug delivery system of H12-(ADP)L551 liposomes could have hemostatic ability in thrombocytopenic animal models (Okamura et al., 2005, 2009, 2010a,b; Tokutomi et al., 2011; Taguchi et al., 2013; Takikawa et al., 2016). The working mechanism of the H12-(ADP)L551 liposomes involves the release of the encapsulated agonist, ADP, in a platelet aggregation-dependent manner to activate circulating platelets while the surface-coated H12-peptides serve as a ligand to bridge activated platelets like Fbg, thereby facilitating platelet aggregates. The H12-(ADP)L551 liposomes are negatively charged and are designed to have a bilayer membrane surface-modified with H12-peptides and 45.2 mol% of dipalmitoylphosphatidylcholine (DPPC), 45.2 mol% of cholesterol, 9 mol% of 1,5-dihexadecyl-*N*-succinyl-L-glutamate (DHSG), 0.3 mol% of 1,2-distearoyl-*sn*-glycerol-3-phosphatidylethanolamine-*N*-[monomethoxypoly(ethyleneglycol)] (PEG-DSPE) and 0.3 mol% of H12-MALPEG-Glu2C_18_; and an aqueous interior encapsulating ADP. As is necessary for the stable repulsion that occurs between platelets in suspension, the electrokinetic charges distributed on the peripheral region of the liposomes are important in allowing them to exist stably *in vitro* and *in vivo* (Seaman and Vassar, 1966). We used the DHSG lipid to provide a negative charge on the liposomal surfaces. This was necessary because from our previous work on red blood cell substitutes and also other reported works, we learnt that negatively charged phospholipids such as phosphatidylserine and phosphatidylglycerol would activate complement proteins and cause coagulation in circulating blood, resulting in a transient reduction in platelet counts and the elevation of the risk of thrombosis (Reinish et al., 1988; Goins et al., 1997; Sakai et al., 2007).

Moreover, we learned that platelet aggregation is the core functional test in platelet studies. Traditionally, light transmission aggregometry (LTA) or turbidimetry is the “gold standard” used in clinical settings. In this method, the conversion of individual platelets to aggregates in suspension is monitored using an attached spectrophotometer which records changes in light transmittance over a period of time. (O'Brien, 1961; Born, 1962). Such an *in vitro* quantification method has been used in clinical settings for the past 50 years. It is widely used for identifying and diagnosing platelet function defects and can be easily performed using commercially available multi-channel aggregometers (Harrison, 2005). Generally, it is advisable that tests are performed within 2 h of blood sampling. However, disadvantages with LTA such as its labor-intensive and time-consuming nature have inspired researchers to devise ways to simulate hemostasis *in vitro* (Michelson, 2004; Harrison, 2005). One such way is the use of a 96-well microplate reader which could cater for a larger sample size with a smaller sample volume in the same assay and measure changes in platelet aggregation in a similar manner as LTA (Bednar et al., 1995).

There is a need to devise a more specific and sensitive method to functionally evaluate nanosized platelet substitutes, such as liposomes. Light transmission aggregometry is unfavorable because it appears to be insensitive to particles smaller than the size of platelets. Optical density is not sensitive to smaller particles as the light scattering intensity from the smaller particles is lower (Ozaki et al., 1994). Moreover, flow cytometry using fluorescently labeled liposomes was also considered; however, the difficulty in training operators and the numerous precise steps required to prepare inactivated platelets in a suitable buffer made testing for aggregation less compelling.

Here, we propose a simple and optimized method which can be used to directly analyze the involvement of liposomes in platelet aggregates using fluorescence imaging microscopy. This method can qualify the presence of platelet aggregates by providing a microscopic view and quantify the *in vitro* aggregation of fluorescently labeled liposomes with activated platelets using image analysis tools. With these two features, we demonstrate that better specificity and sensitivity can be achieved compared with LTA. In the present study, our aims are (i) to determine the optimal washing conditions for aggregated samples, (ii) to determine the optimal final lipid concentration in solution, and (iii) to validate the *in vitro* aggregation of fluorescently labeled liposomes with activated platelets through fluorescence imaging microscopy.

## Materials and Methods

### Materials

Lipid components such as 1,2-dipalmitoyl-*sn*-glycerol-3-phosphocholine (DPPC), cholesterol and 1,5-dihexadecyl-*N*-succinyl-L-glutamate (DHSG) were purchased from Nippon Fine Chemical Co. Ltd. (Osaka, Japan) and 1,2-distearoyl-*sn*-glycerol-3-phosphatidylethanolamine-*N*-[monomethoxypoly(ethyleneglycol)] (PEG-DSPE, 5.1 kDa) was purchased from NOF Co. Ltd. (Tokyo, Japan). Cys-coupled fibrinogen γ-chain dodecapeptide (C-HHLGGAKQAGDV, Cys-H12) was purchased from GL Biochem. (Shanghai, China). The lipid component, H12-MALPEG-Glu2C_18_, where H12 has a cysteine at the N-terminal and is conjugated with a maleimide group at the end of the PEG-lipids, was synthesized in our laboratory (Okamura et al., 2005).

Adenosine 5′-diphosphate sodium salt (ADP), bovine serum albumin, D-(+)-glucose and magnesium chloride, hexahydrate (MgCl_2_.6H_2_O) were purchased from Sigma-Aldrich (St. Louis, MO, USA), while 4-(2-hydroxyethyl)-1-piperazineethanesulfonic acid (HEPES) was purchased from Dojindo Laboratories (Kumamoto, Japan). Calcium (anhydrous) chloride (CaCl_2_), potassium chloride (KCl), sodium citrate, 10% formalin neutral buffer solution and sodium (anhydrous) sulfate (Na_2_SO_4_) were purchased from Wako Pure Chemical Industries Ltd. (Osaka, Japan). Isoflurane, sodium hydrogen carbonate (NaHCO_3_), sodium chloride (NaCl), ethanol (EtOH) and tert-butyl alcohol (t-BuOH) were purchased from Kanto Chemical Co. Inc. (Tokyo, Japan).

### Blood Collection From Guinea Pigs

To eliminate variability from the use of human platelets while developing the assay, guinea pigs from a controlled breeding source were used, resulting in negligible variability between samples and experiments. Considering that a sufficient amount of whole blood was needed to yield enough platelets, we decided to use guinea pigs which are bigger in size. All animal care and experimental procedures were performed in accordance with the guidelines of the Committee for Animal Experimentation at Waseda University. The studies were approved by the Committee for Animal Experimentation at Waseda University (Approval number: 2016-A023). Male Hartley guinea pigs (8 w old and at least 450 g) were housed for at least 1 d before the experiment. They were given free access to water and food prior to a 12 h fast before the experiment. During the experiment, the guinea pigs were firstly anesthetized using 3.5% isoflurane delivered using a gas anesthesia system (PerkinElmer, MA, USA) with an isoflurane vaporizer (Midmark, OH, USA). Subsequently, 2% isoflurane was used for maintenance. Whole blood was collected through a cardiac puncture of the anesthetized guinea pigs.

### Preparation of Platelet-Rich Plasma and Platelet-Poor Plasma

Whole blood (10 mL) was mixed with 1 mL of 3.8% sodium citrate using a 2 mL plastic dropper in an 14 mL polystyrene round-bottom tube (AS ONE, Osaka, Japan). Next, the whole blood sample was centrifuged using a MX-305 centrifuge (Tomy, Tokyo, Japan) at 600 rpm for 15 min at room temperature. Approximately 1 mL of platelet-rich plasma was collected using another plastic dropper and another polystyrene round-bottom tube. Next, platelet-poor plasma was collected after centrifugation of the remaining whole blood at 2000 rpm for 10 min at room temperature. Platelet-rich plasma together with platelet-poor plasma and HEPES - Tyrode buffer (138 mM NaCl, 2.7 mM KCl, 0.40 mM NaH_2_PO_4_, 10 mM HEPES, 12 mM NaHCO_3_, pH 7.4) were used to adjust the final platelet count to 20 × 10^4^/μL as measured using a pocH-100i (Sysmex, Tokyo, Japan).

### Preparation of Fluorescently Labeled Liposomes

Various lipid components were weighed (with reference to 100 mg of DPPC) to achieve the required molar ratio as shown in Table 1 and were dissolved in 6 mL of *tert*-butyl alcohol in a 300 mL round bottom flask. Next, DiD with an approximate molar ratio of 0.05, as shown in Table 1, was added. Sonication at 40°C was performed to obtain a clear blue liquid mixture. Next, freezing with dry ice and acetone was performed to turn the clear blue liquid mixture into a blue ice layer surrounding the internal wall of the flask. The flask was left overnight or for ~20 h to freeze-dry in a Yamato DC400 freeze dryer. The next day, a dry blue mixed lipid powder was collected from the flask and stored stably at −20°C. To prepare a 10 mg/mL liposome dispersion, 50 mg of the powder was dissolved in 5 mL of Dulbecco's phosphate-buffered saline (DPBS). Hydration was performed for 8 h with continuous stirring at room temperature. Next, sequential extrusion (Northern Lipids, Inc, Vancouver, Canada) was performed at room temperature using membrane filters of Φ0.45 μm twice (Millipore), Φ0.22 μm three times (Millipore), and Φ0.20 μm once (Whatman). The use of a final Φ0.20 μm polycarbonate membrane filter with a sharply defined pore size was an added step to ensure that the liposomes would be approximately 200–250 nm in size. Both the particle size and zeta-potential of the liposomes were measured using a Zetasizer Nano (Malvern, UK). The measurement conditions and parameters were as follows: 37°C, dielectric constant 78.5 (for dilute water solution), viscosity 0.8872 cP (for dilute water solution), and the applied voltage was 40 V/cm (for measurement of ζ-potential only). The liposome characteristics are represented as mean diameter ± standard deviation and average ζ-potential ± standard deviation. A Wako phospholipid C measuring kit was used to measure the concentration of liposomes (Wako, Osaka, Japan). To check that the liposomes were equally labeled with DiD fluorescence, we verified the fluorescence intensity of the liposomes in a 10-fold dilution with ethanol. The fluorescence intensity was checked against the fluorescence intensity-concentration curve of DiD and is presented as the concentration of DiD fluorescence ± standard deviation. All measurements were obtained using a spectrofluorophotometer RF-5300PC (Shimadzu, Kyoto, Japan) and assembled using RFPC software for Windows XP. N equals the number of individual experiments and each sample was repeated in triplicate.

**Table 1:** Formulation in molar ratio (mol%) and characterization of liposomes.

**Types of liposomes**	**L550**	**L551**	**H12-L551**
DPPC^a^	5 (49.8%)	5 (45.3%)	5 (45.2%)
Cholesterol	5 (49.8%)	5 (45.3%)	5 (45.2%)
DHSG^b^	0	1 (9.1%)	1 (9.0%)
PEG-DSPE^c^	0.033	0.033	0.033
	(0.3%)	(0.3%)	(0.3%)
H12-MALPEG-Glu2C_18_^d^	0	0	0.033 (0.3%)
DiD^e^	0.045	0.050	0.050
Mean diameter ± SD (nm)	253 ± 94	254 ± 103	246 ± 98
Average ζ-potential ± SD (mV)	−2.8 ± 1.1	−10.2 ± 0.1	−7.5 ± 0.5
Concentration of DiD (μM)	4.1	4.2	4.2

a *1,2-dipalmitoyl-sn-glycero-3-phosphatidylcholine*.

b *1,5-dihexadecyl-N-succinyl-L-glutamate*.

c *1,2-distearoyl-sn-glycero-3phosphatidylethanolamine-n-[monomethoxypoly(ethyl-eneglycol)(5000)*.

d *H12-maleimidyl-n-hydroxy-succinimidyl-ypoly(ethyl-eneglycol)(3400)-n-distearoyl-l-glutamate*.

e *1,1′-dioctadecyl-3,3,3′,3′-tetramethylindodicarbocyanine perchlorate*.

### *In vitro* Aggregation Assay Using a Fluorescence Imaging Microscope

**Figure 1:**
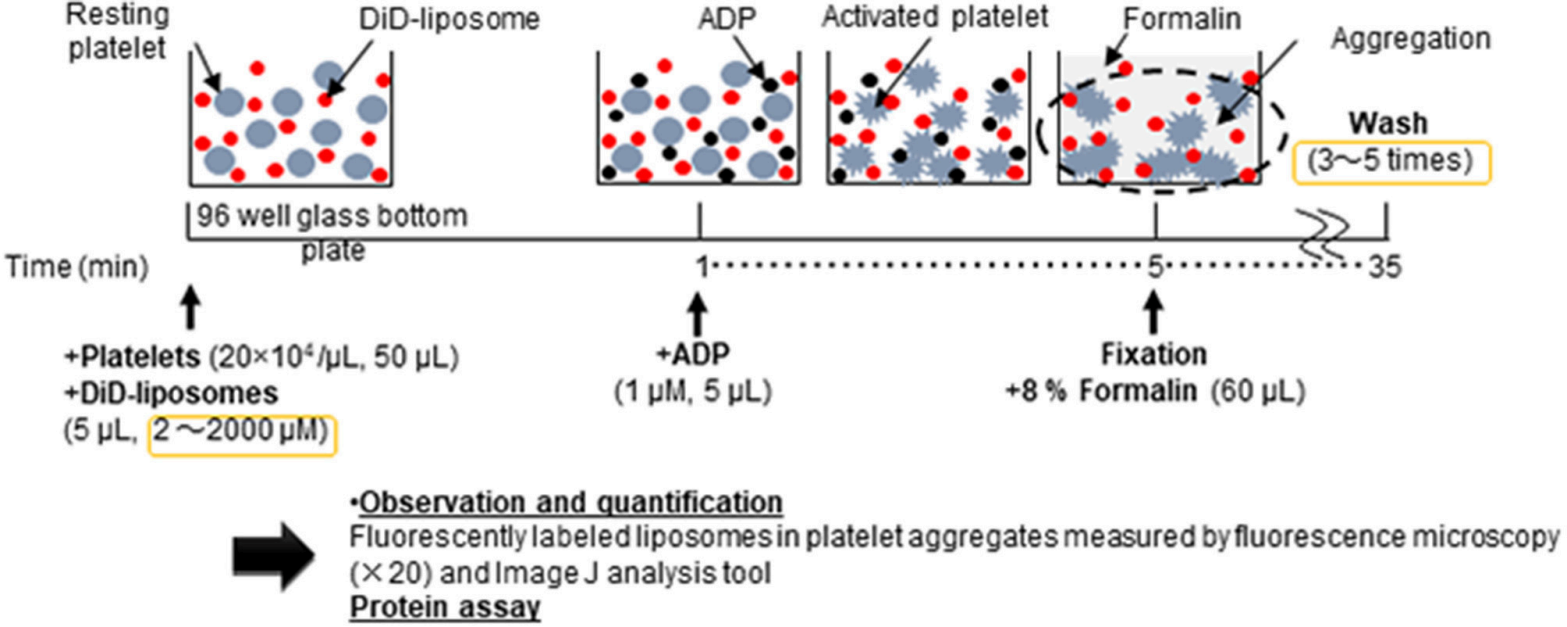
Schematic flow diagram of the *in vitro* aggregation assay of nanosized platelet substitutes with activated platelets by fluorescence microscopy and image analysis.

Figure 1 shows the schematic flow diagram of the *in vitro* aggregation assay. In brief, platelet aggregation was performed in Ezview 96 microtiter glass bottom plates (Iwaki, Tokyo, Japan) in the following manner: 50 μL of 20 × 10^4^ platelets/μL platelet-rich plasma was pipetted into each sample microtiter of the plate. Subsequently, 5 μL of DiD-liposomes (final concentration ranging from 2 μM to 2 mM) was added to each sample per microtiter. The plate was left to equilibrate for 1 min at room temperature in the dark. Next, 5 μL of 1 μM ADP was added into each sample per microtiter (final concentration: 1 μM). The plate was then incubated for aggregation to take place for 4 min at room temperature in the dark. The total volume per microtiter was 60 μL. After incubation, 60 μL of 8% formalin was added to fix each sample for 30 min at room temperature in the dark (final concentration: 4%). After fixation, the samples were washed between three and five times using 100 μL of HEPES-Tyrode buffer through centrifugation using a Hitachi CF16RX centrifuge for 7 min at 1500 × g. After washing, all samples were re-suspended in 100 μL of HEPES-Tyrode buffer. A Thermo Scientific Pierce BCA protein assay was performed in accordance with the manufacturer's protocol (IL, USA). For microscopy purposes, we used the BZ-X710 all-in-one fluorescence microscope (Keyence, Osaka, Japan) and a BZ-X Cy5 filter (excitation: 620/60; emission: 700/75). Raw image files were obtained at 20 × magnification and saved in original resolution. N refers to the number of individual experiments and every sample was tested in triplicate. For quantification purposes, we used the software ImageJ, version 1.50i, to calculate the fluorescence intensity of each sample per microtiter.

### Statistical Analysis

The intensity values calculated using ImageJ were tabulated and reported using Microsoft Excel 2016 with mean ± standard deviation. The data were analyzed using a Kruskal–Wallis ANOVA non-parametric test and a *post-hoc* Steel control test accordingly. A *p* < 0.05 (two-tail) was considered statistically significant.

## Results

### Characterization of Liposomes

Three types of liposomes were prepared, as summarized in Table 1. The L550 liposomes are pegylated liposomes composed of DPPC/cholesterol/PEG-DSPE. The L551 liposomes contain a DHSG lipid which has a carboxyl group as a hydrophilic head group to provide a negative charge on the surface of the liposome. The L551 formulation has also been confirmed to be very stable in aqueous dispersion and in blood circulation (Sakai et al., 1997, 2000; Sou et al., 2000, 2003; Takeoka et al., 2000). The 0.3 mol% of PEG5000-DSPE used in all three types of liposomes means that the surface of the liposomes is not fully covered with PEG chains and forms a mushroom-like conformation (Sou et al., 2007). Therefore, the H12-L551 liposomes which contain 0.3 mol% H12-MALPEG3400-Glu2C_18_ would adequately allow the H12-peptides to be exposed and be recognized by activated platelets (Okamura et al., 2005). The specificity of the H12-peptide has been demonstrated by its non-interaction with resting platelets, and it does not cause platelet activation (Takeoka et al., 2003). The preparation of all liposomes was performed through hydration of the mixed lipid powder in DPBS followed by extrusion through the membrane filters. The sequential extrusion of the liposomes, starting from 0.45 μm and ending with 0.20 μm pore size filters, enabled the mean diameter of the different liposomes to be controlled at approximately 250 nm. The zeta potential values of the L551 and H12-L551 liposomes were more negative than that of the L550 liposomes because of the incorporation of DHSG. The zeta potential value of the H12-L551 liposomes with H12 peptide modification was less negative than that of the L551 liposomes. The difference in zeta potential between the L551 and H12-L551 liposomes possibly reflects the existence of an increased PEG density originating from the H12-MALPEG moiety, as the PEG chains could mask the negative charge of DHSG on the surface of the liposomes. Since the isoelectric point of the H12-peptide is 7.36 and neutral at pH 7.4, the effect of the H12 moiety on the zeta potential would be minimal. The concentration of DiD fluorescence among the three types of liposomes was comparable.

### The Effect of the Number of Washes of the Platelet Aggregates

**Figure 2:**
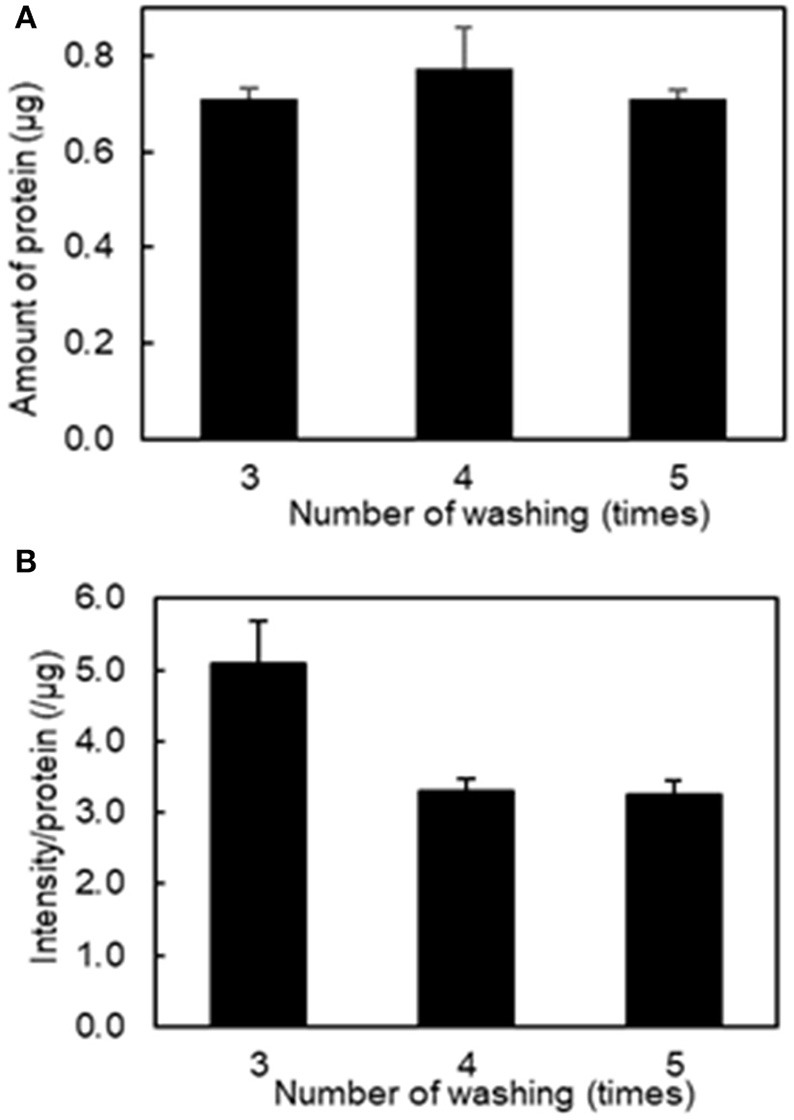
Effect of the number of washing on the amount of protein and fluorescence intensity in the aggregation of H12-L551 liposomes (final concentration: 200 μM) with activated platelets (*n* = 1). **(A)** Amount of protein after washing of aggregated sample between three and five times. **(B)** The mean fluorescence intensity per protein of aggregated sample after washing between three and five times. Error bars indicate SD of three determinations.

To determine the optimal number of washes of the platelet aggregates to remove unbound fluorescently labeled liposomes, we conducted a protein assay and fluorescence imaging of platelet aggregation after washing three to five times. Figure 2A shows that there was no difference in the amount of protein after washing platelet aggregates between three and five times. However, Figure 2B shows that the values of fluorescence intensity/protein in the collected platelet aggregates decreased from 5.08 ± 0.73 μg^−1^ to 3.30 ± 0.20 μg^−1^ after washing three and four times, respectively. This indicates that free liposomes remained after three washes. There was no difference between four and five washes, indicating that the free liposomes were completely washed out after four washes. As a result, we used four washes in the subsequent experiments.

### The Effect of Liposome Concentration

**Figure 3:**
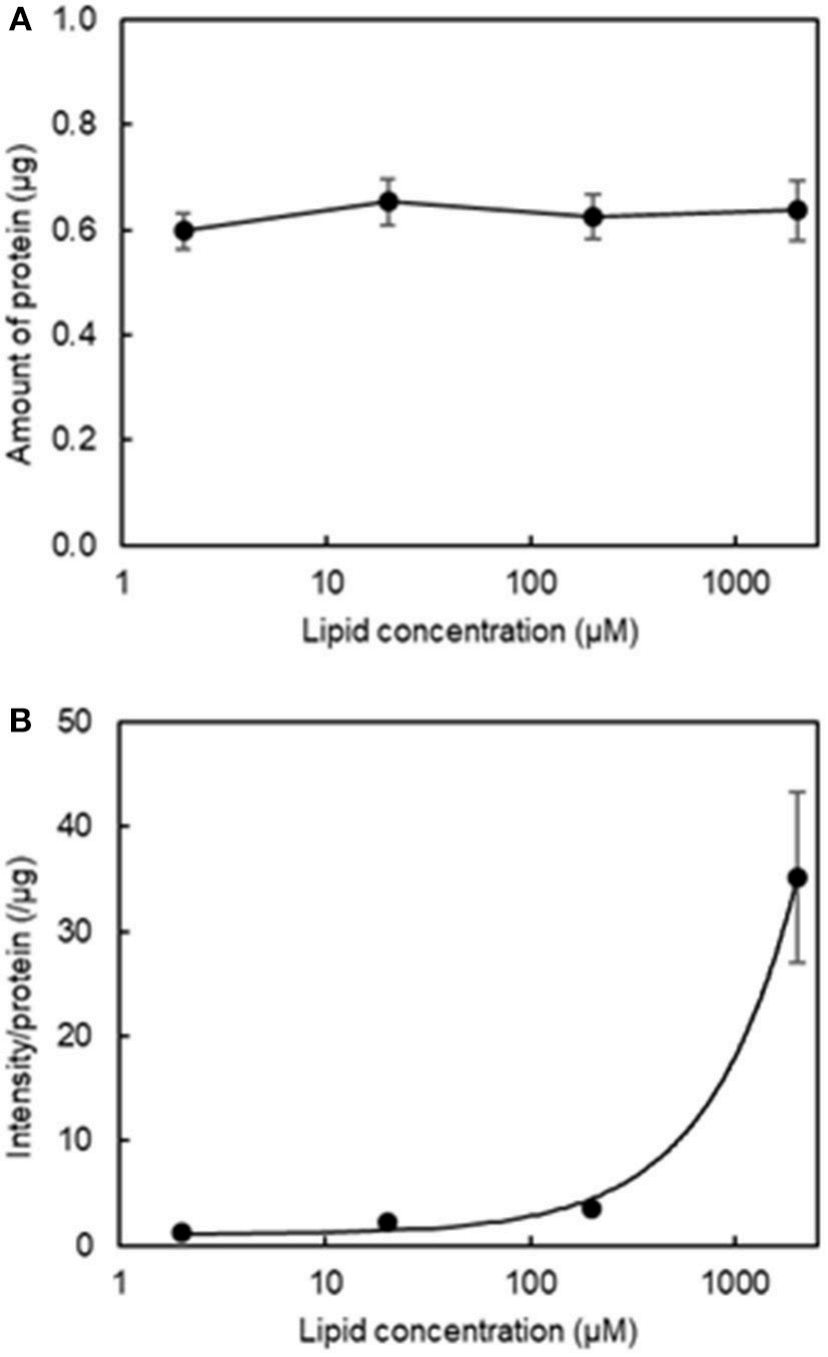
Effect of final lipid concentration on the amount of protein and fluorescence intensity in the aggregation of H12-L551 liposomes with activated platelets (*n* = 1). **(A)** Mean amount of protein at various final lipid concentrations between 2 μM to 2000 μM after washing four times. **(B)** Relationship curve of lipid concentration to mean fluorescence intensity per protein after washing four times. Error bars indicate SD of three determinations.

Our second aim was to determine the optimal lipid concentration for qualitative fluorescence imaging. Figure 3A shows that varying the final lipid concentration between 2 μM and 2 mM with four washes did not result in any changes in the amount of protein and Figure 3B shows the relationship between fluorescence intensity and lipid concentration. We determined a reasonable lipid concentration by assessing the fluorescence images obtained at each concentration. Because of the high sensitivity of the current imaging parameters, the fluorescence intensity may be too high at a higher concentration as it would exceed the upper dynamic range for image analysis. As seen in Figure S1, a lipid concentration of 2 mM was problematic for quantitative image analysis as the fluorescence filled the entire field of view. As a result, we used a final lipid concentration of 200 μM in the subsequent experiments.

### Platelet Aggregation Assessment Using a Fluorescence Imaging Microscope

**Figure 4:**
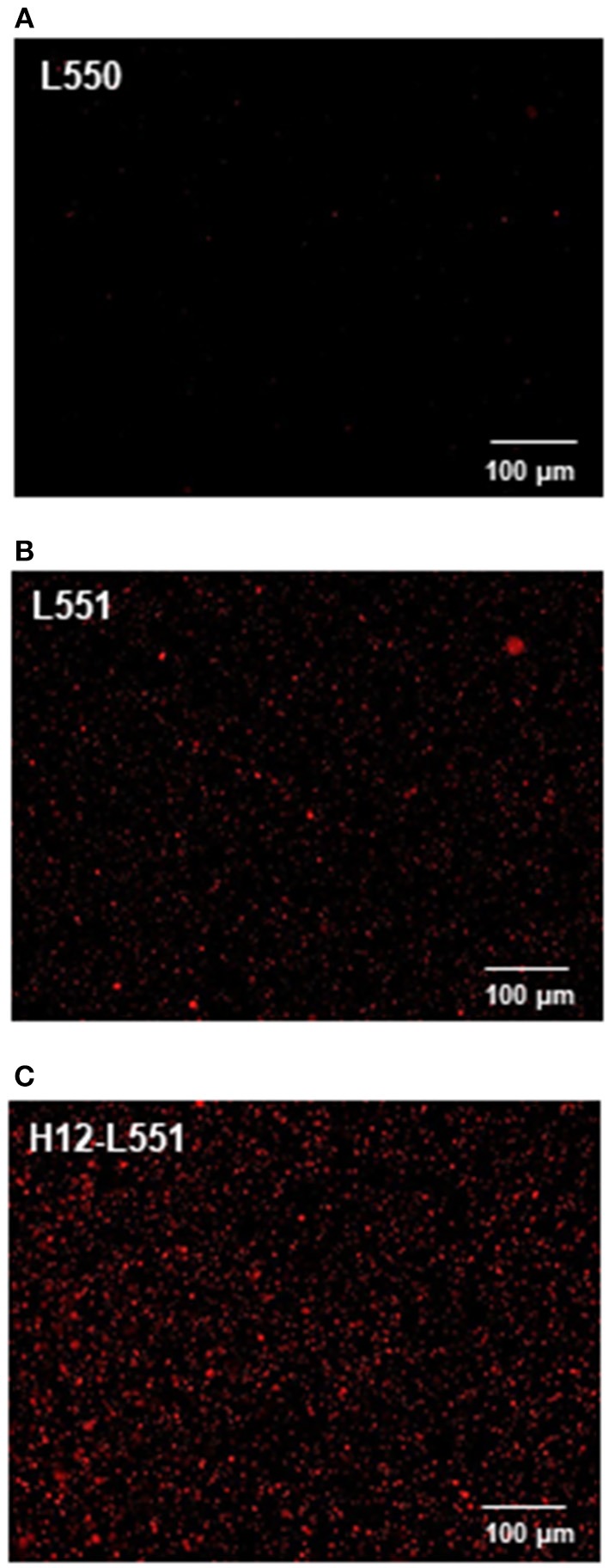
Microscopic images representing the fluorescently labeled unmodified liposomes **(A)** L550, **(B)** L551 and H12-modified liposomes **(C)** H12-L551 in platelet aggregates.

**Figure 5:**
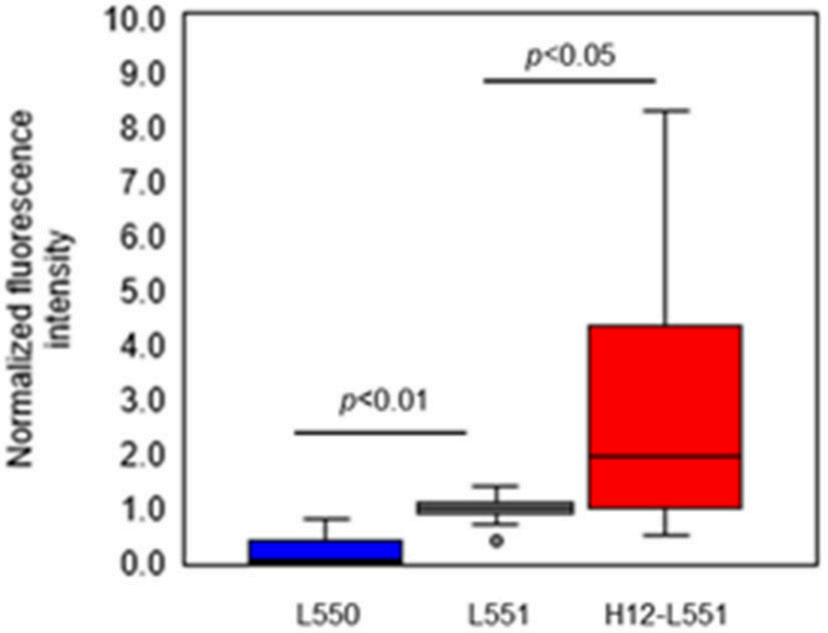
Comparison of normalized fluorescence intensity of fluorescently labeled unmodified liposomes (L550, L551) and H12-modified liposomes (H12-L551) in platelet aggregates after optimizing the conditions (liposome concentration: 200 μM, washing four times). The fluorescence intensity was normalized to mean fluorescence intensity of L551 in each experiment (7 independent experiments with three determinations). Whiskers indicate 0% and 100% quartiles; the boxes indicate 25% and 75% quartiles; the horizontal lines in the boxes indicate the 50% quartile; dot indicates outlier. Comparisons were made by a non-parametric Kruskal-Wallis one-way ANOVA followed by a Steel control test. See Figure S2 for the amount of protein.

Lastly, we performed an *in vitro* aggregation assay of fluorescently labeled liposomes and activated platelets. We added different liposome solutions to an activated platelet dispersion at a final lipid concentration of 200 μM and washed each microtiter sample four times to assess the involvement of liposomes in the platelet aggregates. Figure 4 shows microscopic images of the platelet aggregates in three samples. These images demonstrate that a final liposome concentration of 200 μM is optimal for quantitative image analysis. Figures 4A–C qualify the presence of fluorescently labeled liposomes in increasing order. Figure 4C shows the highest density of fluorescence dots representing H12-L551 liposomes in the platelet aggregates. Figure 5 shows that quantification in the *in vitro* aggregation assay can be achieved using an imaging tool such as ImageJ and this was used throughout the study. A Kruskal–Wallis test was conducted to examine the differences in platelet aggregation based on the type of fluorescently labeled liposomes. Significant differences (Chi-square = 39.415, *p* < 0.05, *df* = 2) were found among the three samples (L550, L551, and H12-L551). The same phenomenon was also observed using human platelets (See Figure S3). A further *post-hoc* Steel control test was conducted to compare two samples. It was shown that there were significant differences between the L550 and L551 liposomes (*p* < 0.01) as well as the H12-L551 and L551 liposomes (*p* < 0.05).

## Discussion

The aim of the current study was to apply a fluorescence imaging technique for the quantitative evaluation of liposomal platelet substitutes involved in platelet aggregates (O'Brien, 1961; Born, 1962; Bednar et al., 1995; Michelson, 2004; Harrison, 2005). Following suitable modifications, this *in vitro* high-throughput aggregation assay has proven to be a specific and sensitive method for the evaluation of the interaction between liposomal platelet substitutes and activated platelets within 2 h of testing using a 96 microtiter plate.

The preparation of fluorescently labeled liposomes was straightforward. We modified a previously reported procedure (Okamura et al., 2005, 2009, 2010a,b) by adding an appropriate fluorescent dye (DiD) into the lipid mixture followed by freeze-drying in the dark. Upon self-assembly during hydration with DPBS, DiD was uniformly inserted in the hydrophobic regions of the lipid membrane. As shown in Table 1, there were no significant differences in the fluorescence intensities between different types of liposomes. A defined platelet concentration of 20 × 10^4^/ μL was adopted from our previously reported works. A lower platelet concentration (data not shown) affects platelet aggregation in most *in vitro* assays. However, the platelet volume used in the current study is merely 20% of the amount typically used for LTA in a 96 microtiter plate (Bednar et al., 1995). This allows more samples to be tested, which would be particularly useful in a clinical setting where demand is higher. A final ADP concentration of 1 μM was also determined through a series of tests (data not shown) and this concentration was sufficient to achieve 50% aggregation, as measured using LTA.

The order and timing of each step, as depicted in the schematic flow diagram (see Figure 1), was carefully studied and this was crucial to the reproducibility and repeatability of this assay. Figure 2 shows that the addition of a suitable fixation reagent was required to stop platelet aggregation during the washing steps to remove noise arising from unbound ADP and fluorescently labeled liposomes (e.g., liposomes not bound to platelet aggregates). We acknowledge that it might be difficult to quantitatively compare the function of different types of nanoparticles, such as micelles and liposomes, based on the fluorescence intensity at a fixed lipid concentration. However, the current method is applicable to determining the half maximal effective concentration using a dose-response curve by adjusting the incorporated amount of the fluorescent dye in the liposomes and changing the imaging parameters. This method can also be used to evaluate receptor blocking experiments or could be further explored for use in comparing the effects of ζ-potential on platelet aggregation.

We have preliminary confirmed that the current method can be used to study human platelets (Figure S3). Our method is sufficiently specific and sensitive for use in a clinical setting. Additionally, it provides a visual aid for users to observe platelet aggregates and assess the differences between unmodified or modified liposomes, as seen in Figure 4. We acknowledge that operator training would be necessary for blood collection and preparation and it is necessary to perform all microscopy analyses within a 2 h time frame. For future use in a clinical setting, this method provides versatility to standardize the guidelines in using platelet substitutes for platelet transfusion. This technique also provides a future perspective in studying platelet activation through using collagen-coated 96 microtiter plates or evaluating the interaction of platelets and liposomes under high shear flow or evaluating ADP encapsulation and release from liposomes. We also acknowledge that in the case of different types of platelet substitutes, which behave differently from liposomes, the assay would need to be reoptimized in the context of the three aims presented here, to ensure that the method is appropriate to evaluate platelet aggregation.

The results shown in Figure 5 confirm that negatively charged liposomes, as well as surface-modified H12-liposomes, are effective in the delivery of encapsulated agonists to activated platelets. The assay presented here allowed us to measure and differentiate between the different types of liposome. Significantly higher amounts of the H12-L551 liposomes were incorporated in the platelet aggregates compared with the L551 liposomes (*p* < 0.05). This is because of a specific interaction between GPIIb/IIIa on the activated platelet membrane and the H12-peptide moiety on the liposomes (Fitzgerald and Phillips, 1985; Rivas and Gonzalez-Rodriguez, 1991; Li et al., 2010). Our previous study demonstrated that H12-L551 liposomes significantly reduce bleeding time compared with L551 liposomes in thrombocytopenic rats (Okamura et al., 2005). Using computed tomography, the accumulation of H12-L551 liposomes at sites of vascular injury has been demonstrated *in vivo* (Okamura et al., 2010a). The correlation between the results in the present aggregation assay and the previous *in vivo* evaluation of the hemostatic effects demonstrate that the present method is sufficiently specific and sensitive to estimate the hemostatic function of liposomes as platelet substitutes. The comparison between the L551 and L550 liposomes revealed that fewer of the L550 liposomes were included in the platelet aggregates compared with the L551 liposomes (*p* < 0.01). This finding suggests that the negatively charged surface, or more specifically DHSG, on the L551 liposomes is involved in their interaction with activated platelets. Further investigation into the effects of DHSG may reveal that it is significant in controlling the interaction between liposomes and activated platelets.

Platelet substitutes could solve the current complications of platelet transfusion systems using donated blood (Lashof-Sullivan et al., 2013; Modery-Pawlowski et al., 2013b). In addition, nanoparticles which specifically bind with activated platelets may have potential applications as drug delivery systems in platelet-relevant diseases (Modery-Pawlowski et al., 2013a; Li et al., 2016). The present *in vitro* high-throughput quantitative aggregation assay facilitates the research and development of platelet substitutes and drug delivery platforms based on nanoparticles. Furthermore, by combining a simple fluorescence imaging microscope with an advanced imaging tool, this method has the potential to evaluate the functional features of *in vitro* primary and secondary hemostasis in a detailed manner.

## Ethics Statement

All animal care and experimental procedures were done in accordance with the guidelines of the Committee for Animal Experimentation at Waseda University. The studies were approved by the Committee for Animal Experimentation at Waseda University (Approval number: 2016-A023).

## Author Contributions

SJT and KN conceived and designed the experiments. SJT, KN, and KS analyzed the data. SJT wrote the first draft of the manuscript. KN, KS, and ST contributed to the writing of the manuscript. KN, KS, and ST agree with manuscript results and conclusions. SJT, KS, and ST jointly developed the structure and arguments for the paper. SJT, KN, KS, and ST made critical revisions and approved the final version.

### Conflict of Interest Statement

The authors declare that the research was conducted in the absence of any commercial or financial relationships that could be construed as a potential conflict of interest.
