# Supplementary material for: An Assay to Evaluate the Function of Liposomal Platelet Substitutes Delivered to Platelet Aggregates
Source: Front Bioeng Biotechnol. 2019 Apr 12;7:77. doi: 10.3389/fbioe.2019.00077 (PMC6473183; doi:10.3389/fbioe.2019.00077)
Supplement: Supplementary file 1 [file Data_Sheet_1.pdf]

An assay to evaluate the function of liposomal platelet substitutes delivered to platelet aggregates

Suyun Janet Tan[1], Keiko Nakahara[1], Keitaro Sou[1,2], Shinji Takeoka[1*]

[1] Department of Life Science and Medical Bioscience, Graduate School of Advanced Science and Engineering, TWIns, Waseda University, Tokyo, Japan

[2] Research Institute for Science and Engineering, Waseda University, Tokyo, Japan

*** Correspondence:**
Takeoka Shinji, PhD
takeoka@waseda.jp

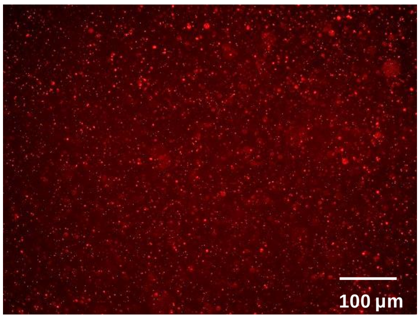

Figure S1. Microscopic images representing the fluorescently labeled H12-modified liposomes, H12-L551, in platelet aggregates and showing that the high lipid concentration of 2mM would cause the fluorescence to cover the entire field of view.

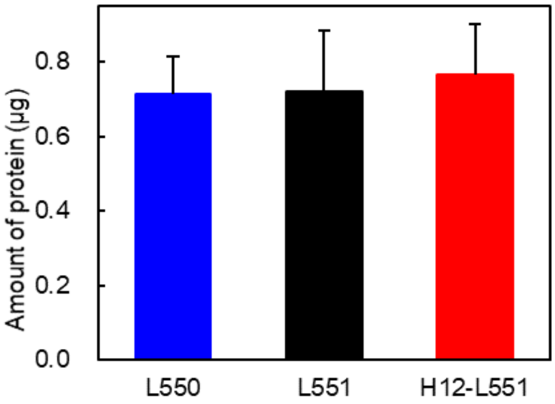

Figure S2. Amount of protein in the aggregation of fluorescently labeled unmodified liposomes (L550 and L551) and H12-modified liposomes (H12-L551) with activated platelets after optimizing the conditions (final liposome concentration: 200 µM, number of washing: four times) (n=7). Error bars indicate SEM of seven independent experiments.

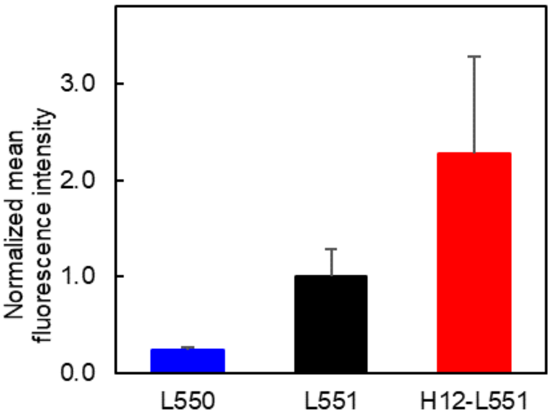

Figure S3. Normalized fluorescence intensity of fluorescently labeled unmodified liposomes (L550, L551) and H12-modified liposomes (H12-L551) in human platelet aggregates after optimizing the conditions (final liposome concentration: 200 µM, number of washing: four times) (n=1). The fluorescence intensity of three determinations was normalized to that of L551. Error bars indicate SD of three determinations.
